# Genome-Wide Identification, Expression Diversication of Dehydrin Gene Family and Characterization of *CaDHN3* in Pepper (*Capsicum annuum* L.)

**DOI:** 10.1371/journal.pone.0161073

**Published:** 2016-08-23

**Authors:** Hua Jing, Chao Li, Fang Ma, Ji-Hui Ma, Abid Khan, Xiao Wang, Li-Yang Zhao, Zhen-Hui Gong, Ru-Gang Chen

**Affiliations:** College of Horticulture, Northwest A&F University, Yangling, Shaanxi, P. R. China; Hainan University, CHINA

## Abstract

Dehydrins (DHNs) play a crucial role in enhancing abiotic stress tolerance in plants. Although DHNs have been identified and characterized in many plants, there is little known about *Capsicum annuum* L., one of the economically important vegetable crops. In this study, seven *CaDHNs* in the pepper genome were identified, which could be divided into two classes: YnSKn- and SKn-type, based on their highly conserved domains. Quantitative real-time PCR (qRT-PCR) results showed that the seven *DHN* genes were expressed in all tissues and might be involved in the growth and development of pepper. The gene expression profiles analysis suggested that most of the *CaDHN* genes were induced by various stresses (low temperature, salt and mannitol) and signaling molecules (ABA, SA and MeJA). Furthermore, the *CaDHN3* (YSK2)-silenced pepper plants showed obvious lower resistance to abiotic stresses (cold, salt and mannitol) than the control plants (TRV2:00). So the *CaDHN3* might act as a positive role in resisting abiotic stresses. This study lays the foundation for further studies into the regulation of their expression under various conditions.

## Introduction

Unfavorable environmental conditions such as cold, high salinity, or drought, limit the growth, development, and distribution of plants which are unable to move away and reduce agricultural productivity [1]. In response to these stresses, plants employ multiple mechanisms to increase their tolerance to various abiotic stresses, such as accumulation of compatible osmolytes (soluble sugars, glycine betaine,and proline) and synthesis of hydrophilic proteins such as dehydrins (DHNs) [2–4].

Dehydrins, group 2 late embryogenesis abundant (LEA) proteins, are expressed during late embryogenesis, as well as in vegetative tissues when subjected to drought, low temperature, high salt and so on [4,5]. There is a positive relationship between the level of accumulation of dehydrin transcripts or proteins and plant stress tolerance. Furthermore, these proteins, a class of unstructured and high hydrophilic proteins, always contain an abundance of charged and polar amino-acids, such as Gly and Pro, and are free of Cys and Trp.

DHNs always are characterized by the conserved Lys-rich 15 amino acid consensus (EKKGIMDKIKEKLPG), known as the K-segment. The K-segment which is the only segment present in all DHNs, is generally present near the C-terminal and can form amphipathic α-helix that may play a role in interaction with membranes and partially denatured proteins [6,7]. Many DHNs include a track of Ser residues named the S-segment, and the Y-segment (DEYGNP) located near the N-terminus [6]. The S-segment can be modified through phosphorylation, which may mediate the nuclear targeting and ion-binding activity such as calcium [8,9]. In addition, nuclear localization signals (NLSs), with an RRKK motif, have been found and also related to the localization of nucleus [10]. According to the number and order of the Y-,S- and K-segment, The DHNs can be devided into 5 subclasses, including YnSKn, YnKn, SKn, Kn and KnS [11].

The dehydrin genes are a multigene family which are distributed not only in higher plants but also in mosses [12,13], algae and cyanobacteria [14,15]. In previous published reports, 10 *DHN* genes had been identified in Arabidopsis [16], 13 in barley [17–19], 8 in rice [20], 6 in tomato [21], 4 in grapevine [22], 12 in Malus domestica [23] and 10 in Poplar [24]. At the functional level, different genes present differential expression profiling throughout development and under various stresses. For example, in barely, 10 *DHNs* were up-regulated by drought, but only 3 were up-regulated by cold [17–19]. Similarly, the expression levels of *DHN1* increased in response to various stresses (drought, cold, heat as well as the application of ABA, SA and MeJA), while neither *DHN3* nor *DHN4* exhibited responsiveness to any of the above treatments in grape [22]. These differences in expression patterns implied functional diversification within these gene families.

In the previously studies, we had cloned and described *CaDHN1* [25]. So far, only a SK3 type dehydrin in pepper was identified [25,26]. Although *DHN* genes have been widely studied in various plants, to date, there is still no comprehensive and systematic characterization of all *DHN* genes in pepper genome. The publication of genome sequence of pepper is convenient to characterize dehydrin gene family in pepper [27,28]. In current study, 7 putative DHN family members from pepper were identified, and their DHN proteins and the expression patterns of *DHN* genes in pepper different tissues and under various stresses were systematically analyzed, aiming to provide a foundation for further functional study and improve the adaptability of pepper to unfavorable growing conditions.

## Materials and Methods

### Identification and chromosome locations of DHN family members from *Capsicum annuum* L.

To obtain all the DHNs in pepper, we used the dehydrin domain sequence (Accession no. PF00257) to find predicted pepper protein sequence data of two pepper cultivar ‘CM334’ and ‘Zunla11’ downloaded from pepper genome database (PGP) (http://peppper.genome.snu.ac.kr/), using The Hidden Markov (HMM) (v3.0) software, which was widely used for identification of homologues of a protein family [29,30]. Meanwhile the published amino acid sequences of DHN members from Arabidopsis [16] and tomato [21] were also used to blast against PGP. To confirm that these obtained sequences encoded dehydrin proteins, the structural analysis of conserved regions were executed by SMART (http://smart.embl-heidelberg.de/) and Pfam (http://pfam.xfam.org/search), and we also examined them for the presence of the highly conserved sequences, a K-segment and its variants. Candidate *CaDHN* genes were aligned with ClustalW2 online software (http://www.ebi.ac.uk/Tools/msa/clustalw2), and the genes with different sequences between the two cultivars were identified. The primer pairs (S1 Table), whose specificity were detected by NCBI Primer BLAST (http://www.ncbi.nlm.nih.gov/tools/primer-blast/index.cgi?LINK_LOC=BlastHome), were designed to amplify these differing quences with Primer Premier 5.0 (Premier Biosoft International, PaloAlto, CA, USA), and CM334 and Zunla-1 sequences for the same gene were then aligned to confirm the correct sequences. The 7 identified DHN proteins were mapped to the chromosomes via MapDraw [31].

### Characterization and comparison of deduced DHN proteins

The deduced pepper amino acid sequences were aligned by Clustal omega online software (http://www.ebi.ac.uk/Tools/msa/clustalo/), and adjusted manually when necessary. Protein MW (molecular weight), pI (isoelectric point), GRAVY (grand average of hydropathy) and composition of amino acids were predicted using Compute pI/MW tool (http://web.expasy.org/computer_pi/). The sequence algorithm NetPhosK (Expasy) was used to predict phosphorylation sites in pepper proteins, with its probability limit set to 60%.

### Phylogenetic analysis, exon/intron struction determination and identification of conserved motifs

The software MEGA 6.0 [32] was used to construct the unrooted phylogenetic trees of the full-length protein sequences, by Neighbor-Joining method [33] and Minimum Evolution method with the parameters (p-distance and completed deletion). Phylogenetic trees were estimated using 1000 bootstraps replicates. The exon/intron construction of the CaDHNs were determined based on alignment of cDNA and pepper genomic sequences using Gene Structure Display Server program (GSDS, http://gsds.cbi.pku.edu.cn/index.php) [34]. Extraction of motif from 7 CaDHN protein sequences were programed using the program of MEME (http://meme-suite.org/tools/meme), with the following parameters: Normal mode, maximum number of motifs was 10 and distribution of motif sites was any number of repetitions.

### Plant materials and treatments

The cold-tolerant pepper cultivar P70 was provided by the pepper research group in Northwest A&F University, China. Pepper seedling were cultivated in a growth chamber (25/20°C day/night temperature and 16/8h day/night photoperiod cycle).

When the 6–8 true leaves expansions period, and growth of about 50 days, pepper seedlings were used for various abiotic stresses and plant hormone treatments. The seedlings of P70 were subjected to 6°C for cold stress, incubated in 300mM NaCl for salt treatment and 300mM mannitol for osmotic stress, and sprayed 0.57mM ABA or 5mM SA, by using the method followed by Guo et al [35]. The spraying of 50μM MeJA were performed as described previously [36]. The leaves were collected at 0, 1, 3, 6, 12 and 24 h after all treatments. To evaluate the expression levels of *CaDHNs* in all tested tissues under normal conditions, six different tissue (roots, stems, leaves, flowers, fruits and seeds) were collected as described previously [25]. All experiments were performed in three biological replicates for each treatment.

### RNA isolation and qRT-PCR analysis

Total RNA was extracted from frozen samples according to the instruction of Total RNA kit (Bio Teke, Beijing, China). Reverse transcription was performed using the Primescript^™^ first strand cDNA Synthesis Kit (TaKaRa, Dalian, China), following the manufacturer’s protocol. The gene encoding the ubiquitin-conjugating protein UBI-3 (GeneBank accession no. AY 486137.1) from pepper was used as the reference gene [37,38]. qRT-PCR was performed in triplicate Using SYBR Premix Ex Taq II (TaKaRa, Dalian, China) on an IQ5.0 Bio-Rad iCycler thermocycler (Bio-Rad, Hercules, CA, USA). Each reaction system was performed as described by Guo et al [35]. The following qPCR reaction systems were used: 95°C for 1min, followed by 45 cycles of 95°C for 15s, 57°C for 20s, and 72°C for 30s. The fluorescence data was collected during the 57°C step. Primers, designed by Primer Premier 5.0, are listed in S2 Table. Relative expression levels of pepper *DHN* genes were determined using the comparative threshold method (2^-ΔΔCt^).

### Virus-induced gene silencing (VIGS) assay of *CaDHN3* in pepper

The pTRV2: *CaDHN3* construct was engineered to include a 443 bp fragment of *CaDHN3* cloned from P70 leaves cDNA template using a gene-specific primer pair (forward primer 5′-AATATGGCACATAACGGTACTAG-3′ and reverse primer 5′- CGGGATCCCTCCAAAGTGATGATGATAAGGT-3′). The underlined nucleotides contained a BamH I restriction site. The resulting PCR product which was sequenced in Quintara Biosciences Company (Wuhan, China), was inserted into pTRV2 vector to form pTRV2:*CaDHN3*. *Agrobacterium tumefaciens* strain GV3101 containing pTRV1 was respectively mixed with pTRV2:00, TRV2-*CaPDS* or TRV2-*CaDHN3* at 1:1 ratio (OD600 = 0.5–0.8 for each construct). The mixtures were injected into pepper leaves, and plants were grown as described by Wang et al [36]. All silencing assays were performed using three replicates, and 50 plants were used for each repetition.

Total chlorophyll content was measured using spectrophotometric method after extracting into 80% (v/v) acetone [35]. Electrolyte leakage was measured according to the method described by Dionisio-Sese and Tobita [39].

### Statistical analysis

All obtained data were subjected to analysis of variance (ANOVA) using SPSS software. The analyzed data were expressed as means ± standard error (SE) of two biological replicates in all measured parameters except for Mn-SOD and POD which were performed using three replicates. The mean separation was analyzed using the Duncan's multiple range test, taking p < 0.05 as a significant difference.

## Results

### Genome-wide identification of *DHN* genes in pepper

The conserved amino acid sequence of dehydrin (Pfam: PF00257) was used to search in the pepper genome database (PGP) (http://pepper.genome.snu.ac.kr/), using the HMMER (v3.0) software. Meanwhile the sequences of DHN members from Arabidopsis [16] and tomato [21] were used to blast against PGP. As a result, a total of 7 candidate *DHN* genes were identified in pepper (Table 1). In addition, providing a simplified nomenclature for each identified gene, we adopted the acronyms of *CaDHN1* to *CaDHN7*, based on their order of appearance from chromosome 1–12. Because a dehydrin gene of pepper had been identified and named to *CaDHN1*, so here, we named the other six genes from *CaDHN2* to *CaDHN7*. The 7 DHNs were then mapped to 3 of 12 pepper chromosomes, with four present on chromosome 2; one on chromosome 4 and two on chromosome 8 (S1 Fig).

**Table 1:** Characteristics of DHN proteins in pepper.

Gene name	Annotation ID	Chr.	Type	Length (aa)	MW (kDa)	PI	GRAVY	SnRK2 No	Ck2 No	PKC No
CaDHN1	CA04g22530	4	SK3	216	24.29	5.41	-1.602	1	6	2
CaDHN2	Capana02g000471▲	2	YSK2	142	15.42	6.66	-1.477	1	1	5
CaDHN3	CA02g06010	2	YSK2	132	13.82	6.43	-1.217	0	3	0
CaDHN4	CA02g22060	2	Y3SK2	172	18.44	7.3	-1.281	1	1	5
CaDHN5	Capana02g002739▲	2	YSK2	170	17.39	6.15	-1.151	1	3	1
CaDHN6	CA00g71940	8	Y3SK2	224	23.04	6.76	-0.885	1	1	10
CaDHN7	CA00g52610	8	SK3	217	24.17	5.57	-1.404	1	6	3

Triangle (▲) marks that sequenced Ids are from Zunla-1 genome, and others with out pentagram from CM334 genome.

### Characterization of deduced DHN proteins

**Fig 1:**
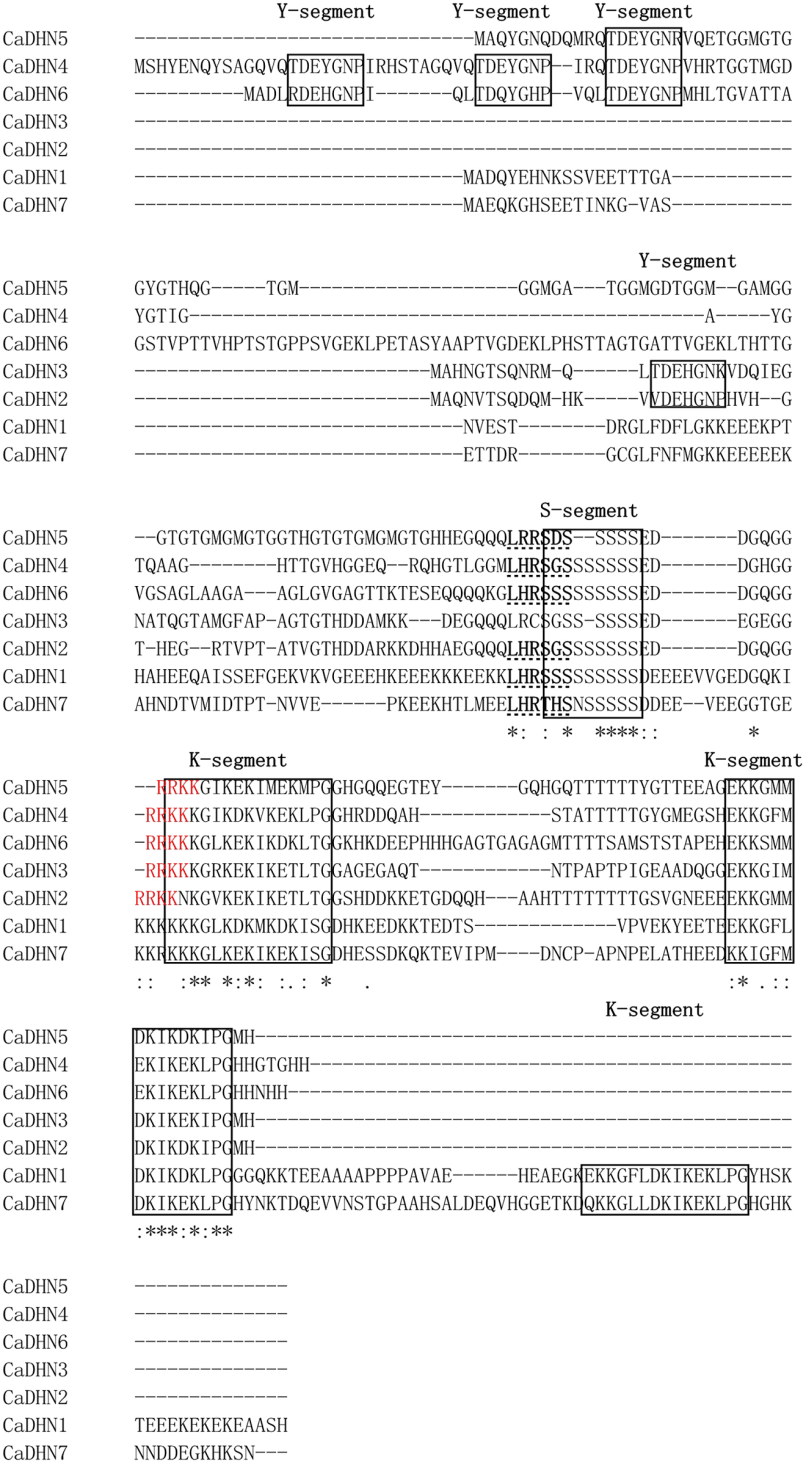
Protein sequence multi-alignment of the DHNs from pepper. S, Y and K are framed by a black line. NLS is highlighted in red font. SnRK-10 sites are in bold and underlined with a dotted line.

Using Clustal omega online software, comparative analysis of the full-length deduced amino acid sequences showed that Y-segment, K-segment and S-segment were found to be highly conserved, but remaining regions displayed relatively low identity among the seven genes. Furthermore, NLSs, with an RRKK motif, were identified in five DHN proteins (CaDHN2, CaDHN3, CaDHN4, CaDHN5, and CaDHN6) (Fig 1). Based on the number and order of Y-, S- and K- motifs, the seven DHNs were classified as YSK2- (CaDHN2, CaDHN3 and CaDHN5), Y3SK2- (CaDHN4 and CaDHN6) and SK3-type (CaDHN1 and CaDHN7) proteins (Fig 1; Table 1).

Generally, DHNs contain an abundance of Gly and polar amino acid, but lack Cys and Trp [9,14]. Analyzing the amino acid compositions of seven DHN proteins, we found that they shared the common feature (S3 Table). The CDS (full-length coding sequence) sizes of 7 CaDHN proteins varied from 399 bp (CaDHN3) to 675 bp (CaDHN6), with respective deduced proteins of 132–224 amino acids in length. The predicted molecular weights were between 13.82 kDa (CaDHN3) and 23.04 kDa (CaDHN6). All members of the CaDHN family were found to be highly hydrophilic, with GRAVY values ranging from -0.885 to -1.602 and pIs (isoelectric point) from 5.41 to 7.30 (Table 1). Among the 7 CaDHN genes, 5 members (CaDHN2, CaDHN3, CaDHN4, CaDHN5 and CaDHN6) that belonged to YnSKn-type, possessed a higher pI than the SKn-type DHN (CaDHN1 and CaDHN7). In addition, we predicted many phosphorylation sites of each DHN protein, with SKn-type (CaDHN1 and CaDHN7) containing a higher number of casein kinase 2 (CK2) phosphorylation sites than putative protein kinase C (PKC) phosphorylation sites, and YnSKn-type containing a higher number of PKC sites than CK2 sites except CaDHN3 and CaDHN5. Beyond that, we also identified a conserved dehydrin motif LXRXXS phosphorylated by an Snf1-related kinase (SnRK2-10) [40].

### Phylogenetic analysis, exon/intron struction determination and identification of conserved motifs

**Fig 2:**
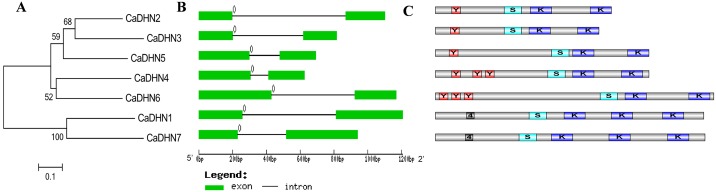
Conserved domains and sequence structure of *CaDHN1* genes. Phylogenetic relationships of CaDHN proteins (A), Exon-intron organizations (B) and conserved motifs (C) of corresponding *CaDHN* genes. Numbers 0, 1 and 2 (B) represent introns in phases 0, 1 and 2, respectively. Each motifs (c) are indicated by a capital or number in the colored box, in which K, Y and S represent K-, Y- and S-segment, respectively, and 4 represents motif-4.

To better understand CaDHN proteins we performed the NJ methods using MEGA 6.0 to obtain the phylogenetic tree based on full-length amino acid sequences of pepper DHN proteins (Fig 2a). The seven CaDHN proteins were classified into three categories: class-I, CaDHN2, CaDHN3 and CaDHN5; class-II, CaDHN4 and CaDHN6; and class-III, CaDHN1 and CaDHN7.

The structures in exon/intron distribution *CaDHN* genes were analyzed based on the alignments of their coding region sequences with respective genomic full-length sequences, and shared a highly conserved exon/intron structures, with one intron and the 0 intron phase (Fig 2b). The length of introns ranged from 106bp (CaDHN4) to 672bp (CaDHN2).

The conserved K-segment, S-segment and Y-segment are also identified as important motifs, which were extracted by MEME based on the CaDHN proteins (Fig 2c). As a result, four significant motifs were obtained (S4 Table). Among these motifs, motif 1 and motif 3 were respectively identified as K- and Y-segments which had high similarities with previous K- and Y-motifs of DHNs. In addition, the motif 2 was identified as S-segment based on a track of Ser residue. The motif 4 was only found in Sk3-type DHNs (CaDHN1 and CaDHN7), which might play a role in the function of the two DHNs (Fig 2c). The distributions of motifs were consistent with the classification of all CaDHN proteins.

**Fig 3:**
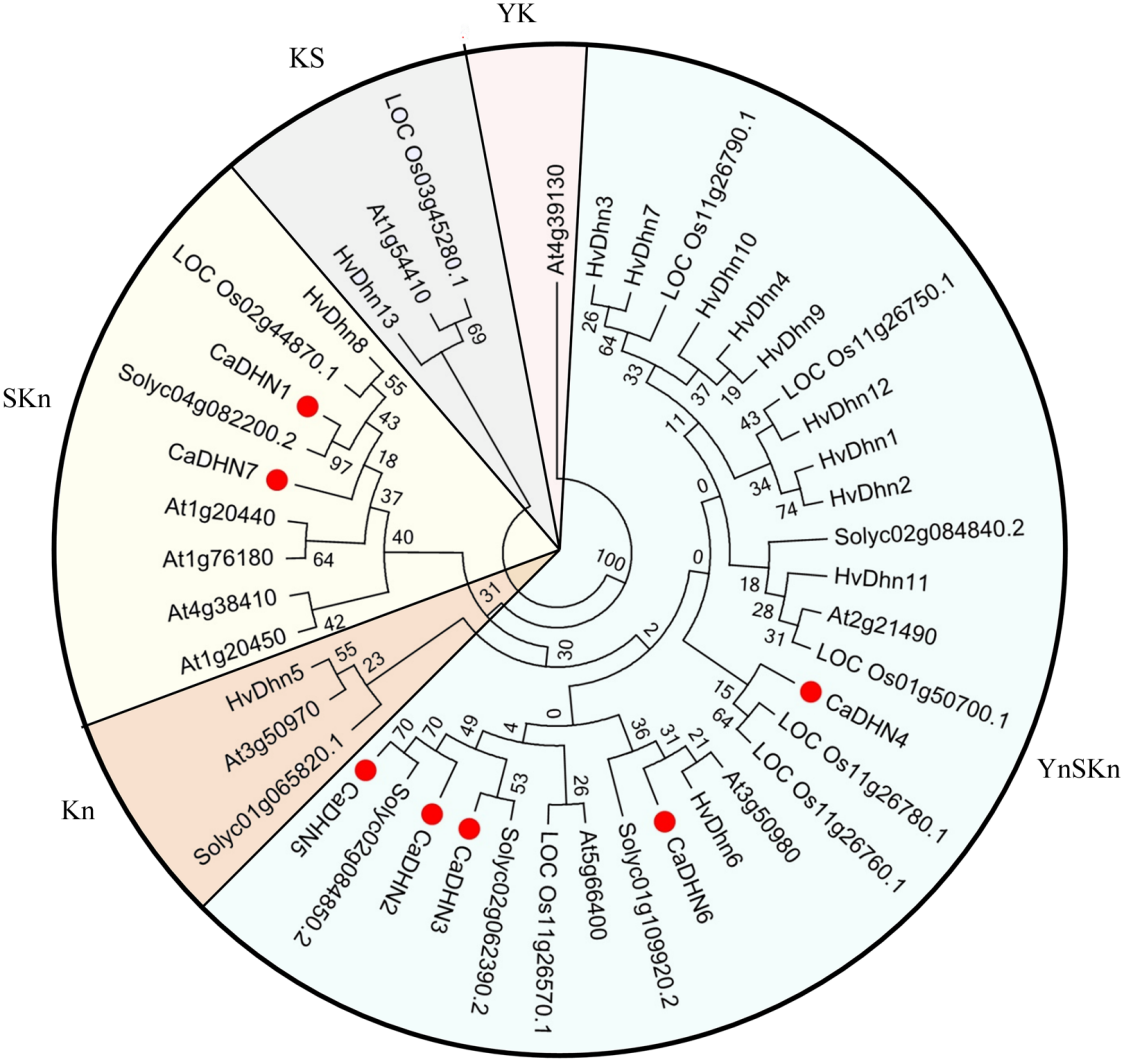
Phylogenetic analysis of dehydrins proteins from pepper (*Capsicum annuum* L., Ca); *Arabidopsis thaliana* (At); barley (*Hordeum vulgare*, Hv); rice (*Oryze sativa*, Os) and tomato (*Solanumly copersicum*, Solyc).

In order to comprehensively analyze the evolutionary relationship between *CaDHN* genes and *DHN* genes from Arabidopsis, rice, barley and tomato, we constructed an unrooted phylogenetic tree of dehydrin proteins. Based on our phylogenetic results, the DHNs could be divided into five groups: YnSKn-, SKn-, Kn-, YK- and KS-type proteins (Fig 3). The classification results of the CaDHN proteins were consistent with the above multiple alignments of CaDHN amino acid sequences (Fig 1), phylogenetic groups(Fig 2a) and the presence of conserved segments(Fig 2c). Interestingly, pepper only had two types and lacked YK-, KS- and Kn-type DHNs. However, more groups are present in Arabidopsis, barley and tomato.

### Expression analysis of *CaDHN* genes in pepper tissues

**Fig 4:**
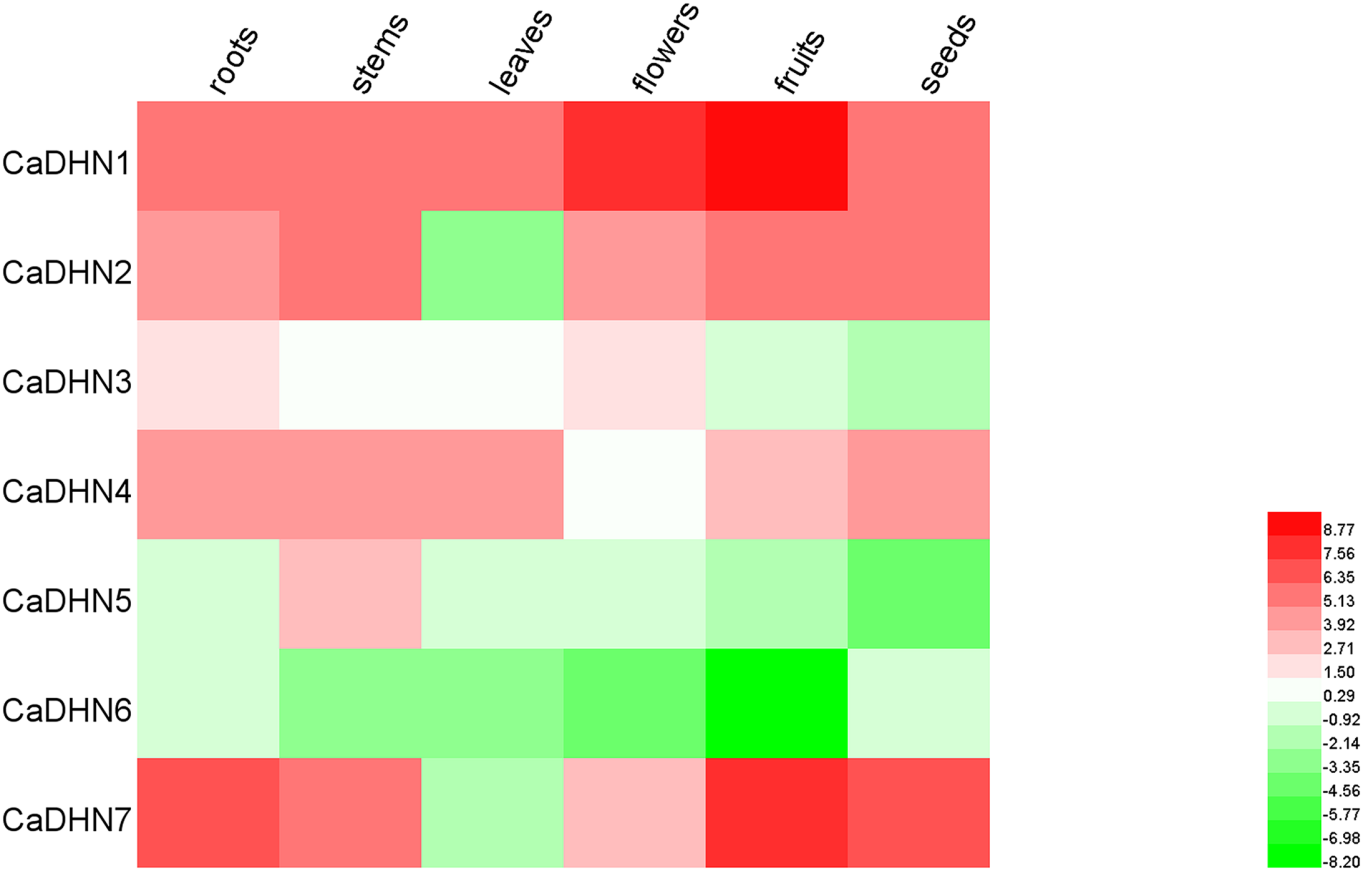
Tissue-specific expression analysis of pepper DHN genes. qRT-PCR data were normalized using the pepper ubiquitin-conjugating proteingene (UBI-3) and are shown relative to the expression levels of roots of the *CaDHN6*.

In order to elucidate the additional information on the functions of different members of DHN family in pepper, the expression of all *CaDHNs* under normal growing conditions was analyzed in six different tissues (roots, stems, leaves, flowers, fruits, and seeds) using qRT-PCR. Expression patterns of the seven pepper *DHN* genes were significantly different. In general, *CaDHN 1*, *2*, *7*, *4* are constitutively expressed in all the tissues, although at different levels and with the lowest expression detectable in the leaves. *CaDHN 3*, *4*, *5* are quiet silent and, perhaps, can be activated at different development or under different conditions (such as stress or hormone treatment). The expression of *CaDHN2* and *CaDHN7* was high in all the tissues, except leaves which were not detected. Concerning the leaves only *CaDHN1* and *CaDHN4* exhibit appreciable expression levels. The expression of *CaDHN1* was at a high level in all tested tissues. However, *CaDHN6* was not detected in any tissue. In addition, we found that the expression of *CaDHN1* and *CaDHN7* (SK3-type), were higher in fruits than other tissues (Fig 4).

### Response of *CaDHN* genes expression to various abiotic stresses

To determine whether *CaDHNs* exhibited stress-responsiveness, we analyzed the expression levels of all genes in the leaves of pepper plants subjected to various stress conditions, using qRT-PCR.

**Fig 5:**
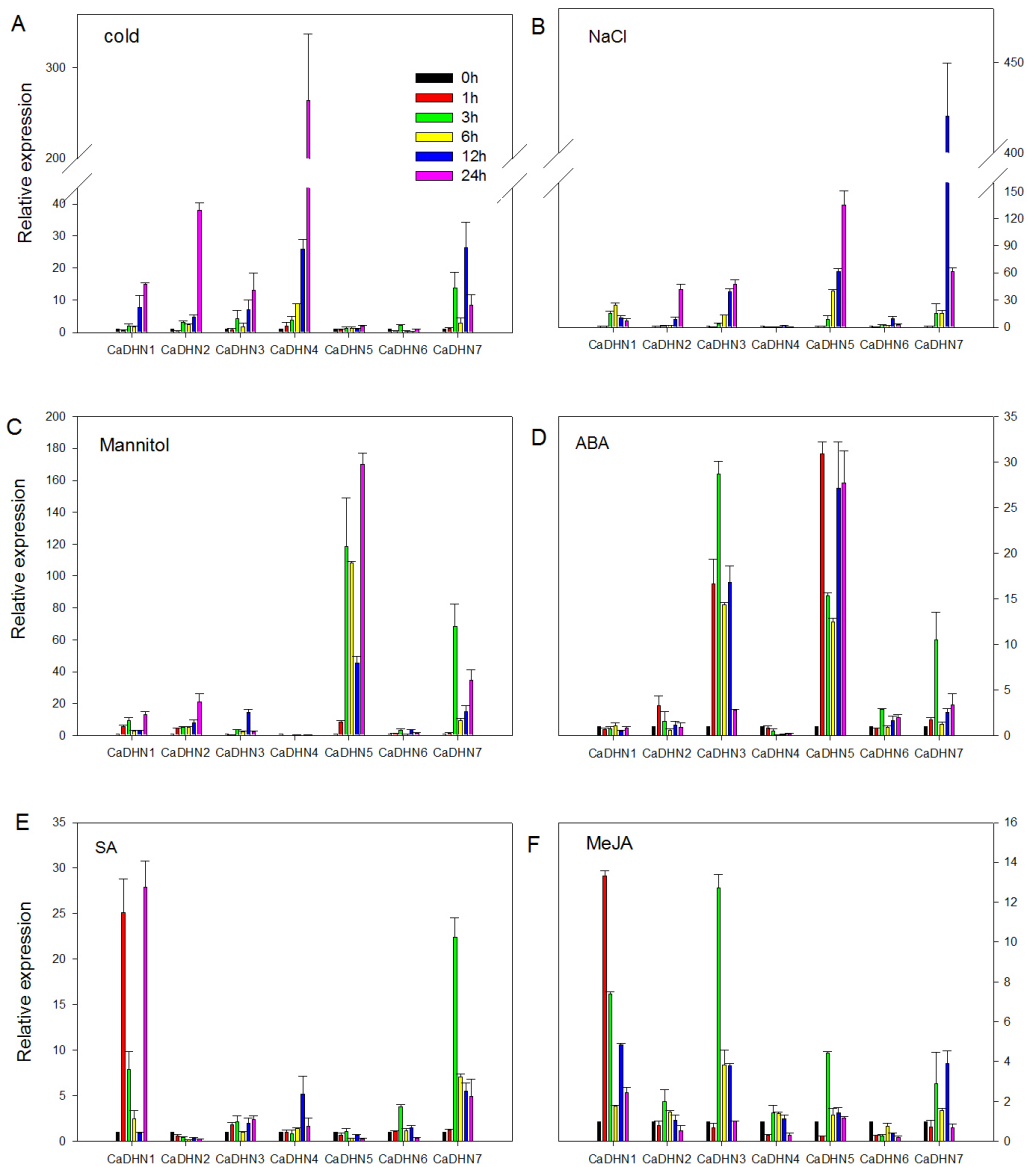
qRT-PCR analysis of *CaDHNs* expression in the leaves of pepper plants following abiotic stresses and plant hormone treatments. The value for each sample is the mean ± standard error (SE), replicated thrice.The expression levels are relative to 0h.

In response to chilling (6°C), the accumulations of mRNA were induced, with levels peaking at 12h (*CaDHN7*) or 24h (*CaDHN1*, *CaDHN2*, *CaDHN3* and *CaDHN4*). Most genes were obviously up-regulated, except *CaDHN5* and *CaDHN6* which were having no obvious change petterns, while *CaDHN4* was especially increased 260-fold at 24h of cold stress (Fig 5a).

Under salt stress treatment, expression of *CaDHN5* and *CaDHN7* increased 134-fold and 420-fold respectively. In addition, the expression of *CaDHN1*, *CaDHN2*, and *CaDHN3* increased by more than 20-fold. Compared with the above genes, the increase multiples levels of *CaDHN6* was smaller. Whereas, *CaDHN4* was down-regulated (Fig 5b).

Under osmotic stress treatment, *CaDHN5* and *CaDHN7* were rapidly up-regulated to 169- and 68-fold, respectively. Other genes, except *CaDHN4* that was down-regulated, were up-regulated more or less (Fig 5c).

### Expression of *CaDHN* genes in response to various signaling molecules

Abscisic acid (ABA) and salicylic acid (SA)/jasmonic acid (JA) generally mediate the responses of plants to abiotic and biotic stress, respectively. So we investigated DHN expression in pepper leaves treated with ABA, SA or MeJA, respectively, aiming to explore whether the response of pepper *DHNs* under stress conditions was related to these molecules. Results showed that by exogenous ABA treatment, the majority of *DHN* genes were significantly up-regulated, especially *CaDHN3* and *CaDHN5*. The exceptions were *CaDHN1* and *CaDHN4*—the former exhibiting not appreciably altered and the latter being down-regulated (Fig 5d). For SA application, all the genes were up-regulated, especially previous research on *CaDHN1* that was strongly induced, with the exception of *CaDHN2* and *CaDHN5*, which showed relatively stable in their expression (Fig 5e). Finally, after MeJA application, most genes were rapidly up-regulated, especially *CaDHN1* and *CaDHN3* increased by more than 10-fold. Whereas the exception was *CaDHN6*, which showed slightly down-regulation (Fig 5f).

### *CaDHN3*-silenced pepper plants reduces tolerance to abiotic stresses

**Fig 6:**
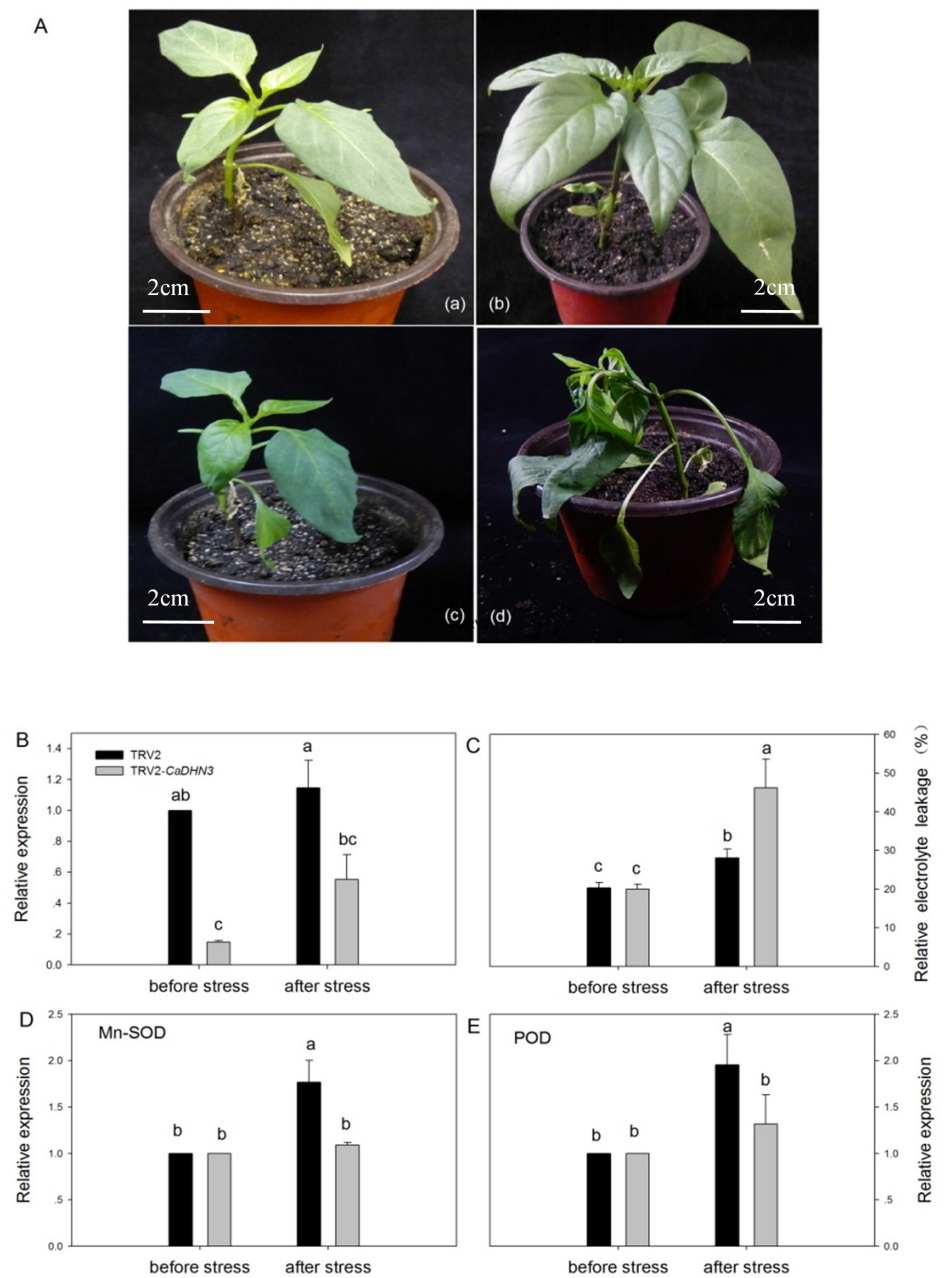
*CaDHN3*-silenced pepper plants. (A) Phenotypes analysis of the *CaDHN3*-silenced and control pepper seedlings under normal growth and 6°C cold stress for 24h. (a,c), control (pTRV2:00) under normal growth and 6°C cold stress for 24h, respectively; (b,d), *CaDHN3*-silenced (pTRV2:*CaDHN3*) under normal growth and 6°C cold stress for 24h, respectively. (B) The expression of *CaDHN3* in gene-silenced pepper (TRV2: *CaDHN3*) and control plants (TRV2:00) were tested at 45 days after inoculation. (C) Effects of 24h of low temperature stress on relative electrolyte leakage in *CaDHN3*-silenced pepper seedlings. (D,E) The expression levels of antioxidant system-relative genes (*Mn-SOD* and *POD*) were assessed in control and *CaDHN3*:silenced plants subjected to low tempreture by qRT-PCR. The results are the mean ± standard error (SE), replicated thrice.

*CaDHN3*, a YSK2-type dehydrin and 132bp amino acids in length, was up-regulated under all of the tested abiotic and hormone treatments. In addition, previous studies showed that YSK2-type dehydrins played an important in enhancing stress resistance [10,11]. Because of the above results, we were interested in the characterization of *CaDHN3*. We studied the function of *CaDHN3*, using the virus induced gene silencing (VIGS) method. When bleaching was evident on the positive control plants (inoculated with TRV2-*PDS*) (S2 Fig), we detected the silencing efficiency with young leaves of *CaDHN3*-silenced plants (inoculated with TRV2-*CaDHN3*) and negative control (inoculated with TRV2). Compared to negative control, the *CaDHN3* silencing rate dramatically reached nearly 85% (Fig 6b) under non-stress conditions. At the same time, the other six genes of pepper family were not silenced (S3 Fig). Whereas the *CaDHN7* was up-regulated to 17-fold. The results showed that there might be a functional interaction between *CaDHN3* and *CaDHN7*. Above all, VIGS was successful and effective for *CaDHN3* gene silencing in pepper.

Under 6°C cold stress for 24h, obvious seriously wilting appeared in *CaDHN3*-silenced plants, while control leaves exhibited slight withering (Fig 6a). In order to confirm the influence of *CaDHN3* silencing in the cold stress defense response, electrical conductivity was measured in control and silenced pepper plants. After 24h of 6°C cold treatments, the electrical conductivity content was significant higher than that of the negative control plants (Fig 6c). In addition, to elucidate the possible mechanism of *CaDHN3* in tolerance to cold stress, the expression levels of *Mn-SOD* and *POD* were examined in control (pTRV2:00) and *CaDHN3*-silenced (pTRV2:*CaDHN3*) plants (Fig 6). The results showed that *Mn-SOD* and *POD* could be cold-induced expression in control plants and *CaDHN3*:silenced plants (Fig 6d and 6e). However, at 24h of cold stress, the expressions of *Mn-SOD* and *POD* genes in *CaDHN3*-silenced plants were significantly lower than in control plants.

**Fig 7:**
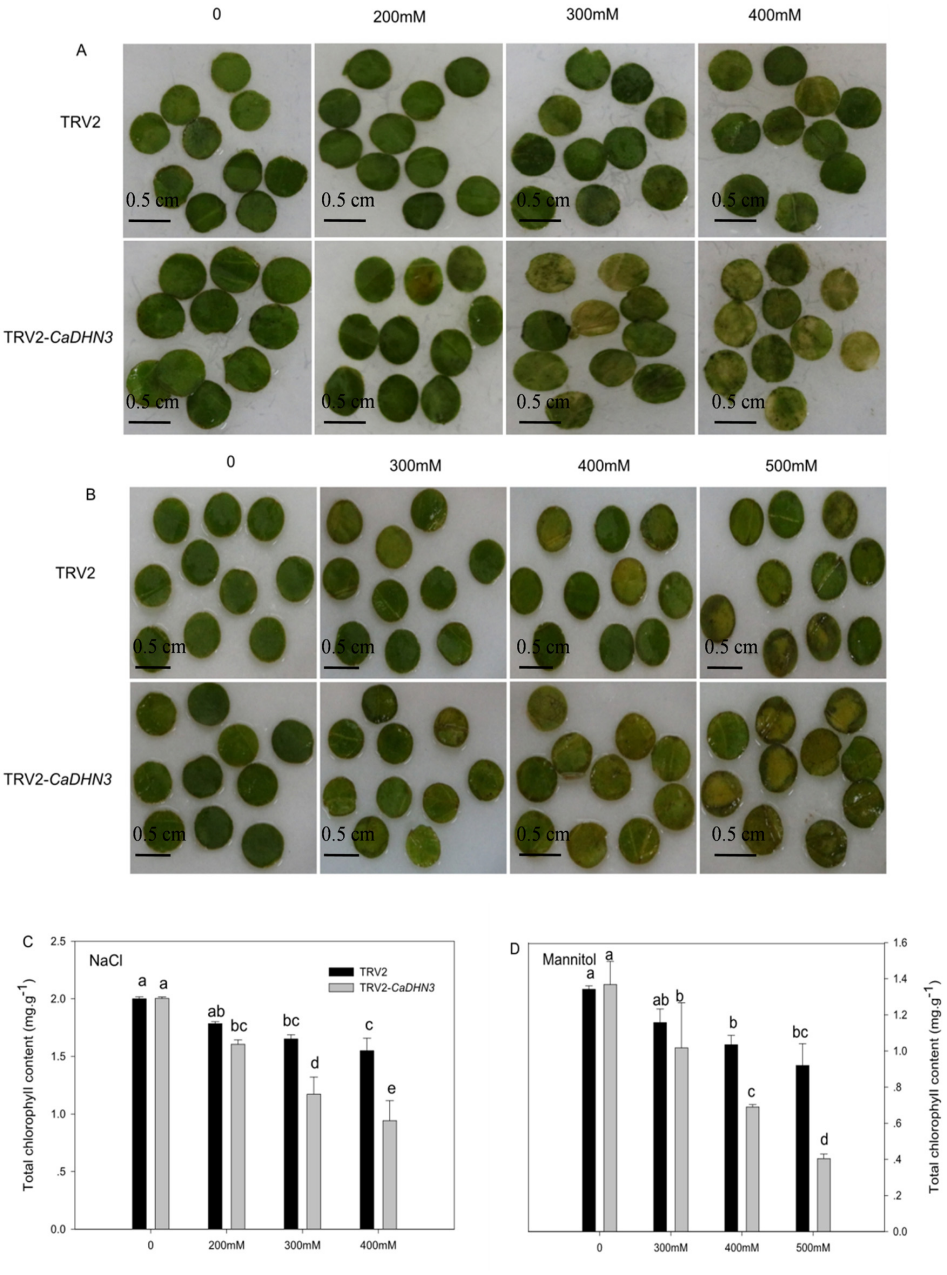
Decreased tolerance of *CaDHN3*-silenced pepper plants to salinity and mannitol stress. Phenotypes of leaf disks in response to salt stress (A) and mannitol stress (B). Chlorophyll content in leaf discs of 3 days after NaCl (C) and mannitol (D) treatments in gene-silenced and control plants.

To determine whether the silencing of *CaDHN3* led to reduced tolerance to salt and osmotic stresses, leaf discs (0.5 cm in diameter) from control (pTRV2:00) and *CaDHN3*-silenced (pTRV2:*CaDHN3*) plants were exposed to different concentrations of NaCl solution (0, 200 mM, 300mM and 400 mM) and mannitol solution (0, 300 mM, 400 mM and 500 mM) respectively with continuous lighting for 3d (Fig 7a and 7b). After 3 days, *CaDHN3*-silenced leaf discs were more yellow and even white symptoms in high concentrations of NaCl solution (300mM and 400 mM) than those of control plants. The same conditions were observed in mannitol-treated plants. The chlorophyll contents of the *CaDHN3*-silenced were also obvious reduced as compared to controls, especially under the stress of high concentrations of salt and mannitol (Fig 7c and 7d).

## Discussion

Dehydrins are believed to play a fundamental role in the response of plants to various abiotic stresses [11,41,42]. They are multigene families and the functions of the dehydrin genes have been characterized in many plants, such as Arabidopsis [16], rice [20], barley [17–19], tomato [21] and so on. Nevertheless, little is known about the pepper DHN family. In this study, a total of 7 DHN family members had been identified in pepper based on the pepper genome (Table 1) [17,28].

Although pepper DHN protein were all highly hydrophilic, they had obvious differences in pI, the number of conserved motifs, CK2 and PKC kinase sites. The pepper YnSKn-type DHNs were higher pI than the SKn-type DHNs. That is, the YnSKn-type DHNs were more easy to combine into negatively charged membrane than SKn-type DHNs [7]. Phosphorylation of the dehydrins may play a role in functional regulation of stressed plant cells and modulate membrane binding of DHNs [43]. SKn-type (CaDHN1 and CaDHN7) contained a higher number of casein kinase 2 (CK2) phosphorylation sites than putative protein kinase C (PKC) phosphorylation sites, and the YnSKn-type (CaDHN2, CaDHN4 and CaDHN6) contained a higher number of PKC sites than CK2 sites except CaDHN3 and CaDHN5. These findings were in agreement with the suggestion of grapevine DHNs family, which YnSKn-type DHNs are mainly phosphorylated by PKC and SKn-type DHNs are mainly phosphorylated by CK2 [4,22,23].

A phylogenetic tree of the DHN family proteins from pepper, Arabidopsis, barley, rice and tomato were constructed. Overall, the members from pepper DHN family except CaDHN4 showed the closer phylogenetic relationship with those of tomato, agreeing with a common ancestor (Fig 3) [28]. The number of pepper dehydrin family was similar to other gene families, such as Arabidopsis and tomato. But it only contained five YnSKn-type DHNs and two SKn-type DHNs, lacked YK-, KS- and Kn-type DHNs which might have been lost in pepper species (Yang et al. 2012). Moreover, the number of the YnSK2-type DHNs (5 in pepper, 3 in Arabidopsis, 8 in barley, 5 in rice and 2 in tomato) accounted for over 50 percent of total DHN members in the phylogenetic tree, making it the largest part. It was consistent with the result that the YnSK2-type DHN proteins were one of the most abundant neutral or alkaline proteins in the nature [11]. The exon/intron structural analysis showed that all of the pepper *DHN* genes contained only one intron and the 0 intron phase (Fig 2b). Guo thought it might be due to a conservative evolution pattern and the length of the insert introns might affect the functional divergences [44].

The expression profiles of *CaDHN* genes were distinct but partially overlapping. Rorat (2006) indicated that DHNs were present in nearly all vegetative tissues during normal growth conditions [11]. Similarly, pepper *DHN* genes expressed in all tissues (roots: 5/7, stems: 5/7, leaves: 2/7, flowers: 4/7, fruits: 4/7 and seeds: 4/7), which suggested that *CaDHN* genes might be involved in the growth and development of pepper. Furthermore, in our study, there was a same conclusion with suggestion that different types of DHNs could localize in the same tissues under the normal growth conditions, for example, *CaDHN2* (YSK2) and *CaDHN7* (SK3) were found to have the same tissue expression patterns. However same types of DHNs could localize in the different tissues under the normal growth conditions. These results could be used to further understand the function of *CaDHN* genes in growth and development of pepper.

*DHN* genes exist as multi-gene families in various plants, and have been well-characterized. Previous studies suggested that SKn and Kn proteins might be largely up-regulated by cold stress, while YnSKn-type DHNs might mainly participate in plant acclimation to salt, ABA and desiccation stresses [11,42]. For example, in barley, almost all of the *DHN* genes encoding for YnSK2 dehydrins were up-regulated by both dehydration and ABA, but not by low temperature. In contrast, *DHN5* (K9) and *DHN8* (Sk3) were up-regulated by cold treatment. Arabidopsis *LTI30* (K6) and wheat *WCS120* (K6) were mainly induced by low temperature stress [45,46]. However, the fact are not invariable. There are large differences among species. Such as, in Ginseng, the majority of *DHN* genes containing various types (SKn-, YnSKn- and KS-type) could be up-regulated by cold, drought and ABA treatment [47]. The *BjDHN1* and *BnDHN1* that belong to Y3SK2-type DHNs, were showed to be up-regulated under cold stress [48]. *MnDHN2* and *MnDHN4* (Y2SK3- and YSK3-type) could be powerful induced by low temperature stress [23]. In our study, seven members of the pepper DHN family exhibited very distinct patterns of expression (Fig 5). *CaDHN1*, *CaDHN2*, *CaDHN3* and *CaDHN7* were induced by cold, NaCl and mannitol stresses. While *CaDHN7* was also highly up-regulated by these treatment, which suggested a possible role in response to abiotic stress in pepper. On the contrary, *CaDHN6* showed no any obvious up-regulation under these stresses. It was worth noting that *CaDHN4* (Y3SK2) was strongly expressed in low temperature conditions and down-regulated by NaCl and Mannitol stresses, which were similar to *MdDHN2* and *MdDHN4* [23]. We believed *CaDHN4* might has an important role in cold condition. Many studies have indicated that plant hormone (ABA, SA and MeJA) play a key role in adaptive environmental stresses and plant developmental processes [49,50]. Dehydrins proteins have an important role in defenses against stress, in either an ABA-dependent or ABA–independent pathway. Most of the *PgDHN* genes and *MnDHN* genes could be induced by ABA [23,47]. The *Physcomitrella patens DHN* gene was induced by ABA [12]. In our experiments, exception for *CaDHN1* and *CaDHN4*, the other pepper *DHN* genes were obvious up-regulated by ABA treatment, which indicated they might be involved in ABA-dependent pathways. These pepper *DHN* genes had different expression pattern in ABA stress. The similar result, Allagulova thought it might be due to the differences of the number of ABA response element in promoter regions [51]. SA and MeJA had been reported to involve in plant stress tolerance, and were important endogenous signaling molecule [36,52,53]. Our results showed that out of seven pepper *DHN* genes, five genes were induced by SA while in case of MeJA six genes were induced.

To determine the function of the *CaDHN3* in abiotic stress responses, we performed VIGS, which is widely used for a rapid detection of pepper genes involved in response to abiotic stresses [54,55]. Previously, we analyzed the function of *CaDHN1*, using the VIGS method [25]. In addition, Chae et al (2015) also analyzed the function of *CaLEA1* gene using this method [56]. In our study, *CaDHN3*-silenced pepper plants exhibited obvious lower resistance to abiotic stresses than the control plants.

In addition, *Mn-SOD* and *POD* were involved in inhibiting the production of reactive oxygen species (ROS) and protecting the cell membrane during the low temperature [57]. *Mn-SOD* gene strongly response to low temperature and oxidative stress reaction [58,59]. Chilling stress enhanced the activities of POD enzymes [59,60]. In our study, we found that the *CaDHN3* gene knockout suppressed the expression of *Mn-SOD* and *POD* (Fig 6), which might be lead to more ROS accumulation in the *CaDHN3*:silenced pepper than in the TRV2:00 pepper plants under stress conditions. These results suggested that *CaDHN3* played an important role in improving pepper stress tolerance and might act as a positive regulator of stress-responsive gene expression.

## Conclusions

In conclusion, we identified 7 *CaDHNs* in the pepper genome. They were divided into two classes (YnSKn- and SKn-type). The transcripts of seven *CaDHN* genes were distinct but partially overlapping expression profiles, and the *DHNs* were expressed in all tissues, suggesting that *CaDHN* genes might be involved in the growth and development of pepper. The gene expression profiles analysis suggested that most of the *CaDHN* genes were induced by various stresses (low temperature, salt and mannitol) and signaling molecules (ABA, SA and MeJA). Further more, the *CaDHN3*-silenced pepper plants showed obvious lower resistance to abiotic stresses (cold, salt and mannitol) than the control plants (TRV2:00). So *CaDHN3* belonging to Y3SK2-type dehydrin, might act as a positive role in resisting abiotic stress. This comprehensive analysis would be an important information for further studies to elucidate the function roles of *CaDHNs* in pepper.

## Supporting Information

S1 Fig Mapping of dehydrin genes family members on pepper chromosomes. Size of chromosome indicated as relative length, the bottom marker indicated each chromosome sequence size.
(TIF)

S2 Fig Phenotypes analysis of the *CaPDS*-silenced pepper seedlings under normal growth at 45 days after inoculation. (TIF)

S3 Fig The expression of *CaDHN* genes in gene-silenced pepper (TRV2: *CaDHN3*) and control plants (TRV2:00) were tested at 45 days after inoculation. The results are the mean ± standard error (SE), replicated thrice.
(TIF)

S1 Table Primers for amplifying the different sequences between CM334 and Zunla-1 genome among DHN members in pepper. (DOCX)

S2 Table Primer sequences used for qRT-PCR analysis. Primers were designed by Primer Premier 5.0, and their specificity was checked by NCBI Primer BLAST. Ubiquitin binding protein gene *UBI-3* from pepper was used as the reference gene.
(DOCX)

S3 Table The contents of amino acids in CaDHN proteins. (DOCX)

S4 Table Motif sequences identified by MEME tools. (DOCX)
