# Supplementary figures and images for: Genome-Wide Identification, Expression Diversication of Dehydrin Gene Family and Characterization of CaDHN3 in Pepper (Capsicum annuum L.)
Source: PLoS One. 2016 Aug 23;11(8):e0161073. doi: 10.1371/journal.pone.0161073 (PMC4995003; doi:10.1371/journal.pone.0161073)

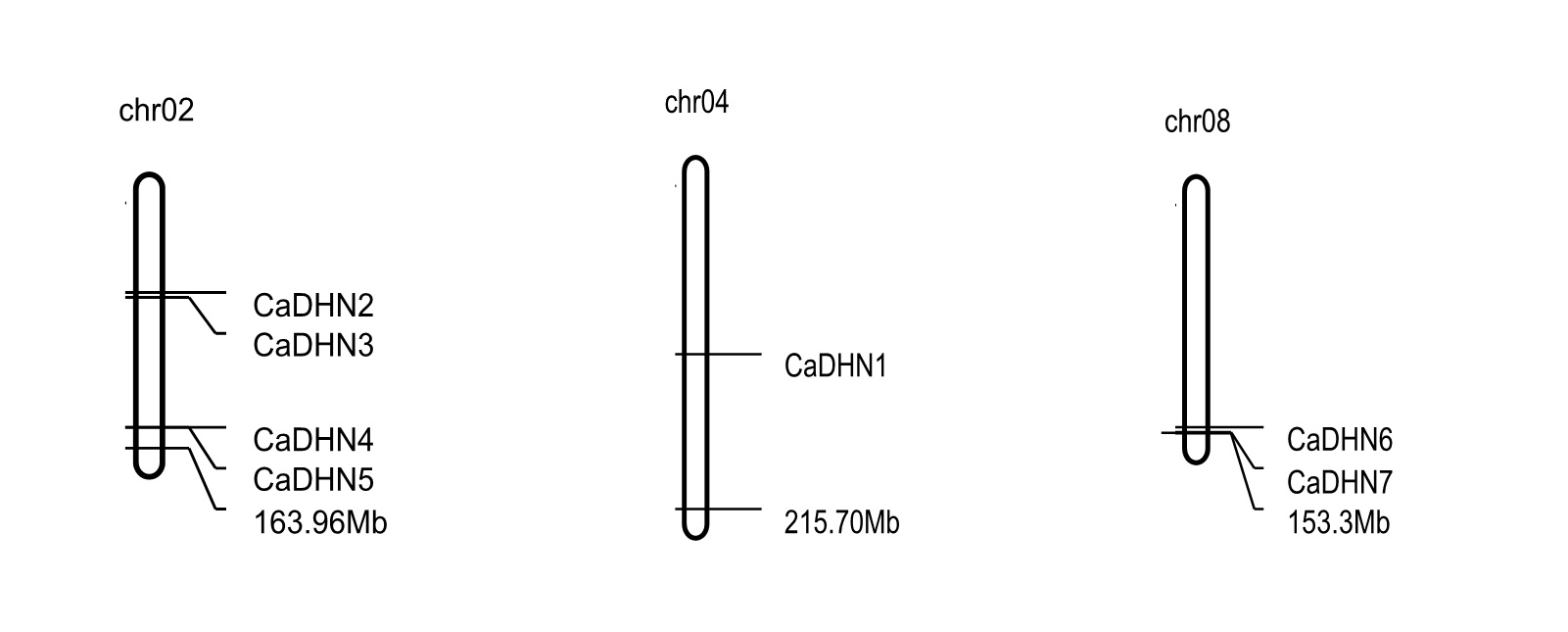

Supplement: S1 Fig — Size of chromosome indicated as relative length, the bottom marker indicated each chromosome sequence size. (TIF) [file pone.0161073.s001.tif]

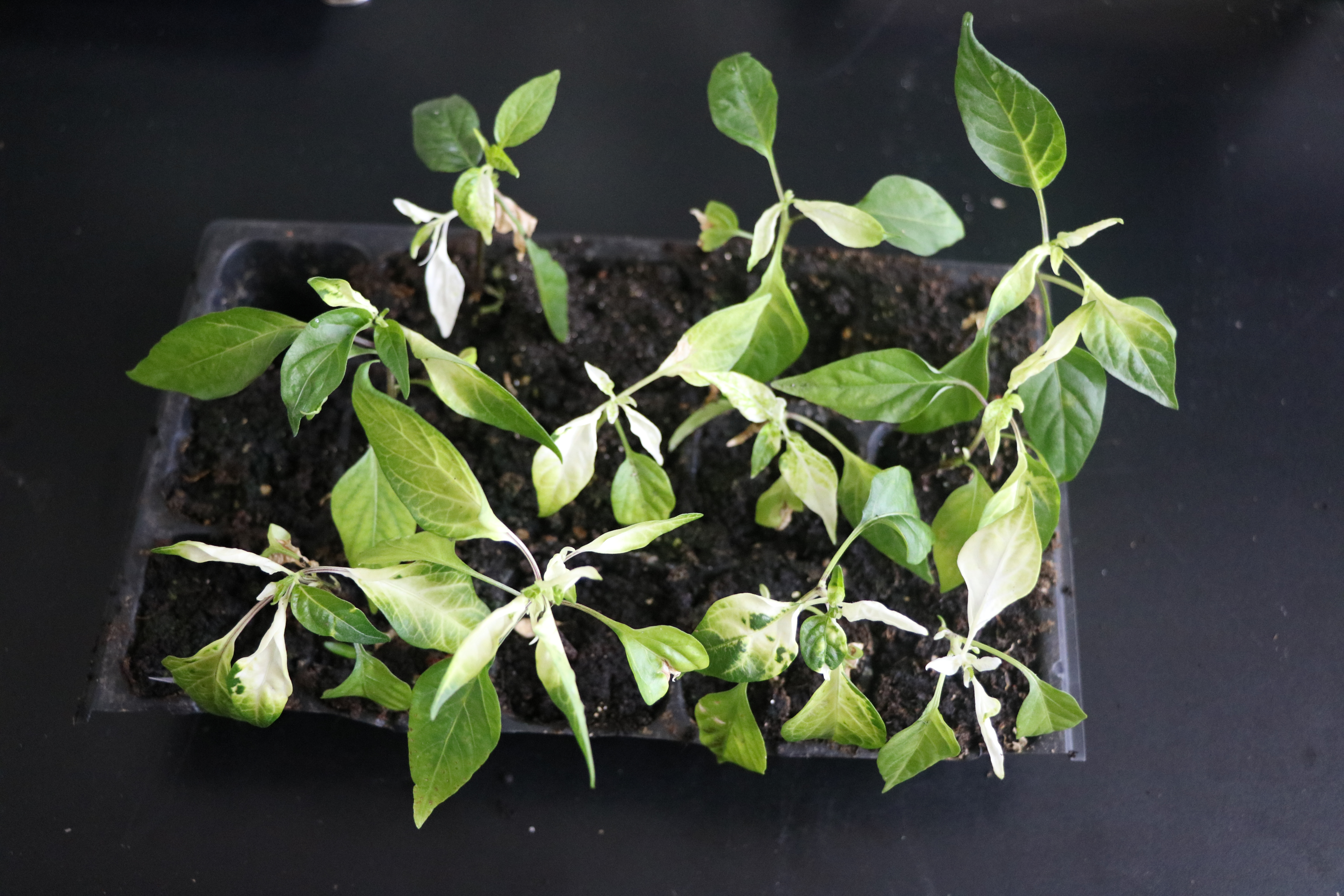

Supplement: S2 Fig — (TIF) [file pone.0161073.s002.tif]

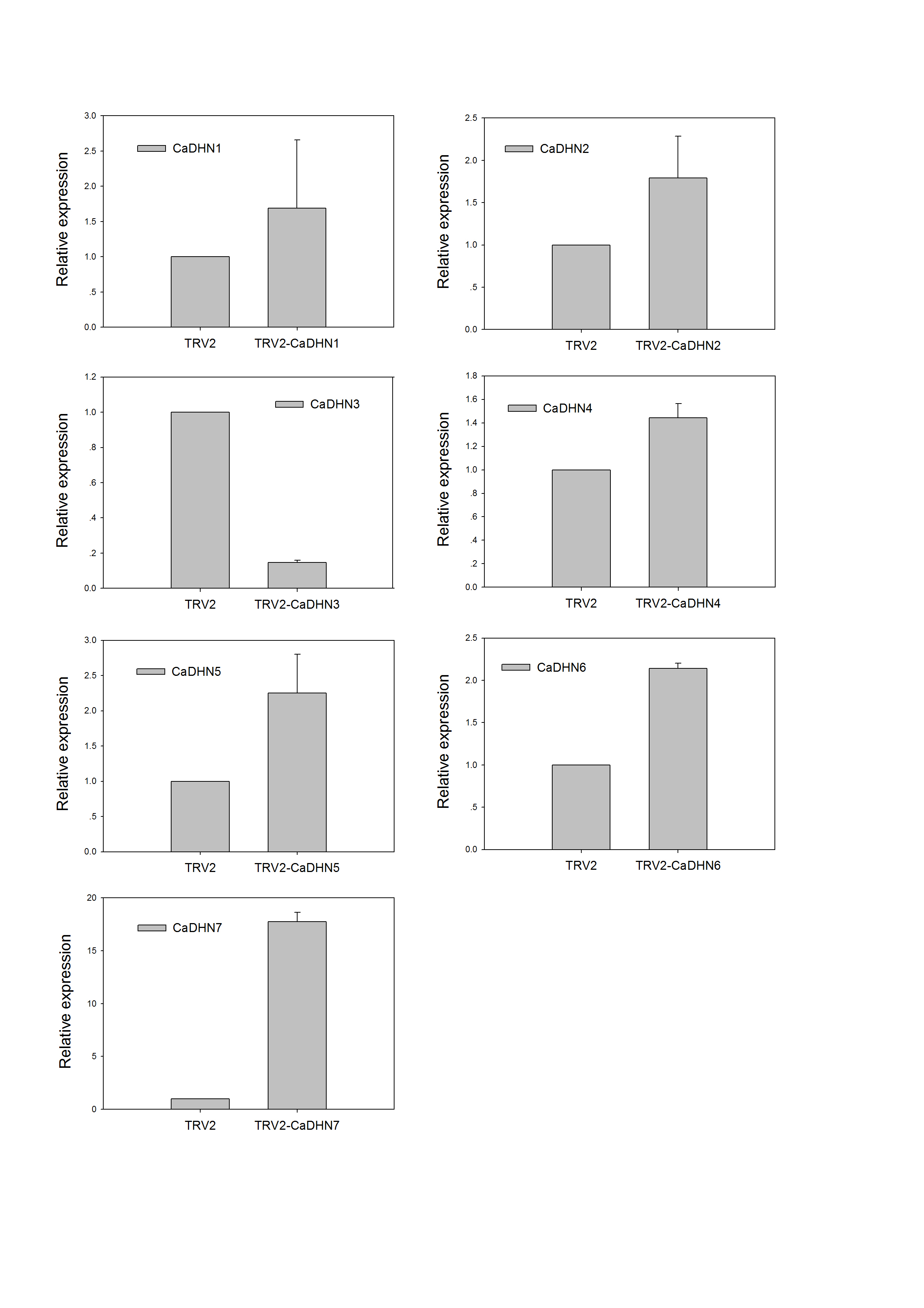

Supplement: S3 Fig — The results are the mean ± standard error (SE), replicated thrice. (TIF) [file pone.0161073.s003.tif]
