# Supplementary material for: Genome-Wide Identification, Expression Diversication of Dehydrin Gene Family and Characterization of CaDHN3 in Pepper (Capsicum annuum L.)
Source: PLoS One. 2016 Aug 23;11(8):e0161073. doi: 10.1371/journal.pone.0161073 (PMC4995003; doi:10.1371/journal.pone.0161073)
Supplement: S1 Table — (DOCX) [file pone.0161073.s004.docx]

**S1 Table. Primers for amplifying the different sequences between CM334 and Zunla-1 genome among DHN members in pepper.**

| Gene name | Forward primer(5′→3′) | Reverse primer(5′→3′) |
| --- | --- | --- |
| CaDHN2 | TTAGGGGAGAGAAATGGCAC | AAGACTATTGGCTTCAACTCAAG |
| CaDHN5 | AGGAGATGGCACAATACGGT | GCTGAAAATGAAAACAAAGGAT |
