# Supplementary material for: Genome-Wide Identification, Expression Diversication of Dehydrin Gene Family and Characterization of CaDHN3 in Pepper (Capsicum annuum L.)
Source: PLoS One. 2016 Aug 23;11(8):e0161073. doi: 10.1371/journal.pone.0161073 (PMC4995003; doi:10.1371/journal.pone.0161073)
Supplement: S2 Table — Primers were designed by Primer Premier 5.0, and their specificity was checked by NCBI Primer BLAST. Ubiquitin binding protein gene UBI-3 from pepper was used as the reference gene. (DOCX) [file pone.0161073.s005.docx]

**S2 Table. Primer sequences used for qRT-PCR analysis.**

| Gene name | Forward primer(5′→3′) | Reverse primer(5′→3′) |
| --- | --- | --- |
| CaDHN1 | AGTGATCATTCTTTGCTTTATTC | TTAACTTTCTCACCAAACTCAGA |
| CaDHN2 | CGTTACAAGCCAAGACCAAAT | CCCTCAGCATGGTGATCTTTC |
| CaDHN3 | ATGGCACATAACGGTACTAGCC | CCCTTCATCTTTCTTCATAGCAT |
| CaDHN4 | TGTCGCACTACGAGAACCAATAT | TCCCTCCAGTACGATGGACTG |
| CaDHN5 | GGCACAATACGGTAACCAAGAC | GTGCCAGTACCTCCACCCAT |
| CaDHN6 | TGAACATGGAAATCCAATTCAAT | CCGTTTCTGGCAATTTTTCA |
| CaDHN7 | ACGCCGACCAATGTGGTAG | TTTTTGCTTATCACTGCTTTCAT |
| Mn-SOD | CTCTGCCATAGACACCAACTT | CCAAGTTCGGTCCTTTAATAA |
| POD | GCAGCATTCCTCCTCCTACT | ATTTCTTTGCCTTGTTGTTG |
| UBI-3 | TGTCCATCTGCTCTCTGTTG | CACCCCAAGCACAATAAGAC |

Primers were designed by Primer Premier 5.0, and their specificity was checked by NCBI Primer BLAST. Ubiquitin binding protein gene *UBI-3* from pepper was used as the reference gene.
