# Supplementary material for: Genome-Wide Identification, Expression Diversication of Dehydrin Gene Family and Characterization of CaDHN3 in Pepper (Capsicum annuum L.)
Source: PLoS One. 2016 Aug 23;11(8):e0161073. doi: 10.1371/journal.pone.0161073 (PMC4995003; doi:10.1371/journal.pone.0161073)
Supplement: S3 Table — (DOCX) [file pone.0161073.s006.docx]

**S3 Table. The contents of amino acids in CaDHN proteins.**

| Amino acid (%) | CaDHN1 | CaDHN2 | CaDHN3 | CaDHN4 | CaDHN5 | CaDHN6 | CaDHN7 |
| --- | --- | --- | --- | --- | --- | --- | --- |
| Ala (A) | 6.0 | 4.2 | 8.3 | 4.1 | 2.4 | 8.5 | 3.7 |
| Arg (R) | 0.9 | 3.5 | 3.8 | 4.7 | 3.5 | 1.8 | 1.4 |
| Asn (N) | 0.9 | 2.8 | 3.8 | 2.3 | 1.2 | 1.3 | 5.5 |
| Asp (D) | 6.0 | 8.5 | 6.1 | 5.2 | 4.7 | 4.0 | 7.4 |
| Cys (C) | 0.0 | 0.0 | 0.8 | 0.0 | 0.0 | 0.0 | 0.9 |
| Gln (Q) | 1.9 | 6.3 | 6.8 | 5.8 | 6.5 | 3.6 | 2.3 |
| Glu (E) | 20.8 | 7.7 | 9.1 | 6.4 | 8.2 | 7.1 | 14.7 |
| Gly (G) | 7.4 | 12.0 | 16.7 | 8.7 | 28.8 | 14.3 | 8.8 |
| His (H) | 4.2 | 9.2 | 3.0 | 18.6 | 4.1 | 7.1 | 6.0 |
| Ile (I) | 2.3 | 2.1 | 4.5 | 2.9 | 2.4 | 1.3 | 3.7 |
| Leu (L) | 3.7 | 1.4 | 2.3 | 2.3 | 0.6 | 8.5 | 4.6 |
| Lys (K) | 20.4 | 11.3 | 10.6 | 7.0 | 5.9 | 5.8 | 15.2 |
| Met (M) | 0.9 | 0.0 | 4.5 | 2.9 | 10.0 | 2.7 | 2.8 |
| Phe (F) | 2.3 | 3.5 | 0.8 | 0.6 | 0.0 | 0.0 | 1.4 |
| Pro (P) | 3.7 | 2.1 | 3.8 | 2.9 | 1.2 | 5.8 | 4.1 |
| Ser (S) | 8.3 | 7.7 | 6.1 | 10.5 | 3.5 | 8.9 | 6.5 |
| Thr (T) | 4.6 | 12.0 | 8.3 | 7.6 | 13.5 | 13.8 | 6.5 |
| Trp (W) | 0.0 | 0.0 | 0.0 | 0.0 | 0.0 | 0.0 | 0.0 |
| Tyr (Y) | 1.4 | 0.0 | 0.0 | 4.7 | 2.9 | 1.3 | 0.5 |
| Val (V) | 4.2 | 5.6 | 0.8 | 2.9 | 0.6 | 4.0 | 4.1 |
| Pyl (O) | 0.0 | 0.0 | 0.0 | 0.0 | 0.0 | 0.0 | 0.0 |
| Sec (U) | 0.0 | 0.0 | 0.0 | 0.0 | 0.0 | 0.0 | 0.0 |
