# Supplementary material for: Genome-Wide Identification, Expression Diversication of Dehydrin Gene Family and Characterization of CaDHN3 in Pepper (Capsicum annuum L.)
Source: PLoS One. 2016 Aug 23;11(8):e0161073. doi: 10.1371/journal.pone.0161073 (PMC4995003; doi:10.1371/journal.pone.0161073)
Supplement: S4 Table — (DOCX) [file pone.0161073.s007.docx]

**S4 Table. Motif sequences identified by MEME tools.**

| Motif | Width | Multilevel consensus sequence |
| --- | --- | --- |
| 1 | 18 | REKKGFMDKIKEKLPGMH |
| 2 | 15 | LHRCSSSSSSSSDDD |
| 3 | 8 | LTDEYGNP |
| 4 | 7 | CGLFDFL |
